# High-dose versus standard-dose amoxicillin/clavulanate for clinically-diagnosed acute bacterial sinusitis: A randomized clinical trial

**DOI:** 10.1371/journal.pone.0196734

**Published:** 2018-05-08

**Authors:** Andrea Matho, Mary Mulqueen, Miyuki Tanino, Aaron Quidort, Jesse Cheung, Jennifer Pollard, Julieta Rodriguez, Supraja Swamy, Brittany Tayler, Gina Garrison, Ashar Ata, Paul Sorum

**Affiliations:** 1 Albany Medical Center Hospital, Albany, NY, United States of America; 2 Albany Medical College, Albany, NY, United States of America; 3 Albany College of Pharmacy and Health Sciences, Albany, NY, United States of America; 4 Department of Surgery, Albany Medical College, Albany, NY, United States of America; 5 Departments of Medicine and Pediatrics, Albany Medical College, Albany, NY, United States of America; Tongji Hospital of Tongji Medical College of Huazhong University of Science and Technology, CHINA

## Abstract

**Background:**

The recommended treatment for acute bacterial sinusitis in adults, amoxicillin with clavulanate, provides only modest benefit.

**Objective:**

To see if a higher dose of amoxicillin will lead to more rapid improvement.

**Design, setting, and participants:**

Double-blind randomized trial in which, from November 2014 through February 2017, we enrolled 315 adult outpatients diagnosed with acute sinusitis in accordance with Infectious Disease Society of America guidelines.

**Interventions:**

Standard-dose (SD) immediate-release (IR) amoxicillin/clavulanate 875 /125 mg (n = 159) vs. high-dose (HD) (n = 156). The original HD formulation, 2000 mg of extended-release (ER) amoxicillin with 125 mg of IR clavulanate twice a day, became unavailable half way through the study. The IRB then approved a revised protocol after patient 180 to provide 1750 mg of IR amoxicillin twice a day in the HD formulation and to compare Time Period 1 (ER) with Time Period 2 (IR).

**Main measure:**

The primary outcome was the percentage in each group reporting a major improvement—defined as a global assessment of sinusitis symptoms as “a lot better” or “no symptoms”—after 3 days of treatment.

**Key results:**

Major improvement after 3 days was reported during Period 1 by 38.8% of ER HD versus 37.9% of SD patients (P = 0.91) and during Period 2 by 52.4% of IR HD versus 34.4% of SD patients, an effect size of 18% (95% CI 0.75 to 35%, P = 0.04). No significant differences in efficacy were seen at Day 10. The major side effect, severe diarrhea at Day 3, was reported during Period 1 by 7.4% of HD and 5.7% of SD patients (P = 0.66) and during Period 2 by 15.8% of HD and 4.8% of SD patients (P = 0.048).

**Conclusions:**

Adults with clinically diagnosed acute bacterial sinusitis were more likely to improve rapidly when treated with IR HD than with SD but not when treated with ER HD. They were also more likely to suffer severe diarrhea. Further study is needed to confirm these findings.

**Trial registration:**

ClinicalTrials.gov Identifier: NCT02340000.

## Introduction

Acute sinusitis is typically treated with antibiotics even though most studies have shown little benefit [1]. These studies are, however, hard to interpret because they used different inclusion criteria, antibiotic regimens, and outcome measures. To maximize the benefit of treatment, the Infectious Disease Society of America (IDSA) recommends in its 2012 guidelines [2] that clinicians use strict criteria to diagnose acute bacterial sinusitis and prescribe amoxicillin/clavulanate at the standard dose (SD) of 875 /125 mg bid for 7 days as first-line treatment (unless the patient is allergic to penicillin). It recommends using the high dose (HD) of 2000 /125 mg bid if the rate of penicillin-resistant *Streptococcus pneumoniae* in the community is greater than 10%. The available HD is a pharmacokinetically-enhanced extended-release (ER) tablet of amoxicillin/clavulanate that aims to achieve a longer duration of therapeutic amoxicillin concentrations by providing ER amoxicillin along with immediate-release (IR) clavulanate [3].

Few clinicians will, however, know the level of penicillin resistance in their communities. Furthermore, higher concentrations of amoxicillin in the sinuses may help in treating penicillin-sensitive, as well as penicillin-resistant, pneumococci and other bacteria. First, in purulent secretions, the low pO2 and high pCO2 may impair antibiotic effectiveness [4]. Moreover, the diffusion of amoxicillin into closed spaces is difficult and may become increasingly difficult as membranes thicken during the illness [5]. For middle ear infections in children, this line of reasoning was confirmed by aspirates of middle ear fluid and by clinical experience [6–8] and led to the adoption in 2004 of HD amoxicillin as the standard of care [9,10]. Its applicability to acute sinusitis is suggested by the finding that, in patients with acute sinusitis, nasopharyngeal cultures showed eradication of 4 of 5 penicillin-sensitive *S*. *pneumoniae* with HD amoxicillin/clavulanate but only 2 of 6 with SD [11]. Studies of amoxicillin and clavulanate concentrations in sinus tissue itself have, however, involved only patients with chronic sinus problems [12–15].

Second, studies in children and adults of standard doses of amoxicillin [16,17] and even of amoxicillin/clavulanate [18,19] have demonstrated little or no clinically significant benefit. In contrast, among children aged 1–10 years (41% > 6), 50% of those receiving HD amoxicillin/clavulanate were cured at 14 days versus only 14% receiving placebo [20]. Adults with acute sinusitis would not be expected to differ fundamentally from school-age children in their response to antibiotics. Accordingly, even though HD amoxicillin/clavunate is not superior to SD in adults with community-acquired pneumonia [3,21] or bronchitis [22] (or only slightly so [23], it may provide added benefit in adults with acute sinusitis.

A further issue of importance is what outcome to measure. The first question is what measure to use. Most studies of acute sinusitis have measured changes over time in patients’ current assessment of a set of symptoms [16,17,20], not their global assessment of improvement [24]. Some health researchers argue, however, that the latter is more valid [25]. Indeed, overall ratings are common in clinical medicine and simple for patients, and improvement is what patients and clinicians want. The second question is when to make the primary measurement. Most studies of acute sinusitis have focused on improvement at 7–15 days [1,16,19,26], not at 3–4 days [17]. Studies of common infectious diseases have shown, however, that, while most people eventually get better, treatment—for example, antimicrobials for Streptococcal pharyngitis [27], influenza [28], or acute otitis media [10]—can decrease the severity and duration of illness, even if only by a day or two. These short-term outcomes can be important to patients.

The purpose of this study was to determine if, in an outpatient setting with a suspected low prevalence of penicillin-resistant pneumococci, the early as well as late clinical benefits of HD amoxicillin/clavulanate, as assessed primarily by a global rating of improvement, are greater than its detriments when compared to SD.

## Methods

We conducted this randomized, double-blinded, comparative-effectiveness, pragmatic [29] trial from November 18, 2014, through March 29, February 2017 in the academic primary care Internal Medicine/Pediatrics practice of Albany Medical College in Latham, New York (see S1 Table: Consort Checklist). The study protocol was approved by the Institutional Review Board (IRB) at Albany Medical Center on October 16, 2014 (see S1 Text: Original Protocol). We started the process of registration at ClinicalTrials.gov at the same time as we started recruiting participants, with provisional registration on November 22, 2014; final registration, however, was not completed until January 16, 2015. We confirm that our new trial of the same intervention was registered at ClinicalTrials.gov prior to enrolling any participants. When, midway through the trial, the ER HD formulation became unavailable from the manufacturer, the IRB approved on February 9, 2016, a revised protocol to study the difference in efficacy and tolerability, compared with SD, of 2 different HD formulations (see S2 Text: Revised Protocol). The study was funded by contributions from the corresponding author. Written informed consent was obtained from each participant.

### Participants

Patients age 18 and older who presented to the office with sinus symptoms were eligible for inclusion if not previously enrolled and if, in the judgment of the treating clinician, they fit into one of the three diagnostic categories for acute bacterial sinusitis, as defined by the 2012 IDSA clinical guidelines [2]: 1) persistent symptoms and not improving (lasting for ≥ 10 days); 2) severe symptoms or signs of fever ≥ 102 degrees F and nasal discharge or facial pain (lasting for ≥ 3–4 days); or 3) worsening symptoms or signs characterized by new onset of fever, headache, or increase in nasal discharge following a typical viral URI that lasted 5–6 days and was initially improving (double sickening).

Patients were excluded if they were allergic or intolerant to amoxicillin or amoxicillin/clavulanate; were breastfeeding; were cognitively unable to give informed consent and/or to assess their symptoms; were taking allopurinol; had chronic or recurrent “sinus” problems, defined as persistent “sinus” congestion, not attributed to nasal allergies, for 8 weeks or more [30] or 2 or more episodes of antibiotic-treated “sinusitis” in the past 3 months; were judged by clinicians to need HD amoxicillin/clavulanate, doxycycline, or levofloxacin because of severe infection, treatment with amoxicillin or penicillin within the past month, or immune compromise; needed hospitalization; were suspected to harbor methicillin-resistant *Staphylococcus aureus;* or had a contraindication to amoxicillin/clavulanate because of chronic kidney disease stage 4, hepatic impairment, current mononucleosis, or a history of antibiotic-associated colitis.

### Interventions

Our pharmacist (GG) randomized participants in a 1:1 ratio by preparing and labeling prescription bottles in medication bags with consecutive study numbers containing either SD or HD amoxicillin/clavulanate. The enrolling clinicians, the patients, and the residents and medical students performing follow-up telephone calls were blinded to the dosage (although an unknown number of patients found pictures of their pills on-line).

The participants received 2 bottles of tablets with instructions to take 1 tablet from each bottle twice a day with food for 7 days. The SD participants had 1 bottle with amoxicillin/clavulanate 875/125 mg and the other with placebo tablets (of over-the-counter lactase); the total daily dose of amoxicillin was 1750 mg. The HD participants had tablets of amoxicillin/clavulanate 1000/62.5 mg ER in each bottle until, with patient #180, we had to switch the HD formulation to 1 bottle with amoxicillin/clavulanate 875/125 mg IR and the other with amoxicillin 875 mg IR. The total daily dose of amoxicillin in the HD group thus decreased from 4000 mg to 3500 mg (from 2.3 to 2.0 times that of the SD group) with no change in the dose of clavulanate.

All participants filled out the Sinonasal Outcome Test-16 (SNOT-16), the questionnaire validated by Garbutt and colleagues [17,31,32], on which they rated the severity of 16 symptoms on a scale of 0 = no problem to 4 = severe problem (Table 1).

**Table 1:** SNOT-16 questionnaire^[^^17^^,^^28^^]^. Rate how much you have been bothered, in terms of severity and frequency, by the following symptoms.

Symptoms	No problem	Mild or slight problem	Moderate problem	Severe problem
1. Need to blow nose	0	1	2	3
2. Sneezing	0	1	2	3
3. Runny nose	0	1	2	3
4. Cough	0	1	2	3
5. Postnasal discharge	0	1	2	3
6. Thick nasal discharge	0	1	2	3
7. Ear fullness	0	1	2	3
8. Headache	0	1	2	3
9. Facial pain or pressure	0	1	2	3
10. Wake up at night	0	1	2	3
11. Lack of a good night’s sleep	0	1	2	3
12. Wake up tired	0	1	2	3
13. Fatigue	0	1	2	3
14. Reduced productivity	0	1	2	3
15. Reduced concentration	0	1	2	3
16. Frustrated, restless, or irritable	0	1	2	3
Total score (maximum 48)				

The clinicians performed anterior nasal cultures to look for colonization with penicillin-resistant pneumococci and other pathogens (stopped after participant #231 because of lack of funds), gave participants written suggestions for symptomatic treatment with acetaminophen and nasal saline (as described in S3 Text: Suggestions for Treating Symptoms), and gave them a list of the questions they would be asked at the end of Day 3.

### Outcomes

Participants were called at the end of 3 and 10 days of treatment, using a standardized script, to ask about improvement and adverse effects. Improvement was assessed in 2 ways: by a global rating of sinus symptom improvement on a 6-point scale of 1 = a lot worse, 2 = a little worse, 3 = the same, 4 = a little better, 5 = a lot better, and 6 = no symptoms; and by the responses again to the SNOT-16 questions. This global rating scale had been used by Garbutt and colleagues to validate the SNOT-16 [31] and was a secondary outcome of their clinical trial [17]. Tolerability was assessed by asking participants to rate on a scale of 0 = none to 3 = severe the degree of diarrhea, abdominal pain, vaginal discharge and itching (for women), rash, and other. At Day 10, they were also asked how many doses they did not take and if they would take this antibiotic again.

The primary efficacy outcome was the percent of patients in each group who gave a global rating of 5 or 6 after 3 days of treatment. Day 3 was chosen because the study’s focus was on rapid improvement (as discussed in the Introduction) and because Day 3 was Garbutt and colleagues’ first assessment point [17]. The secondary efficacy outcomes were the percent that gave a global rating of 5 or 6 at Day 10 and the average changes in the ratings on the SNOT-16 questions at Day 3 and Day 10 compared to baseline ratings (with a minimally important difference of 0.5 units [31]).

### Statistical analyses

To determine sample size, we assumed that 34% of the SD group would give a rating of 5 or 6 at the end of Day 3 (the improvement with placebo in Garbutt’s study [17]); decided that an improvement to 50% in the HD group (an effect size of 16%) would be clinically significant; and calculated a need for 292 patients to provide an 80% power to detect this amount of improvement with an alpha of 0.05 [33].

Using SPSS version 24 (IBM SPSS), we performed chi-square analyses, using a two-tailed P of <0.05 to determine significance, to compare baseline characteristics, global symptom ratings, adverse effects after 3 and 10 days, and percent declaring at Day 10 that they would not take the antibiotic again. We performed t-tests to compare changes in SNOT-16 scores. We did these analyses first for the study as a whole and then for each time period. In the latter analyses, we compared each of the two HD formulations only to the SD participants during the same time period to reduce confounding by differences in other factors, such as circulating viruses, pollen counts, or weather.

## Results

For the full data, see S1 Dataset: Sinusitis Study.

### Patient characteristics

**Fig 1:**
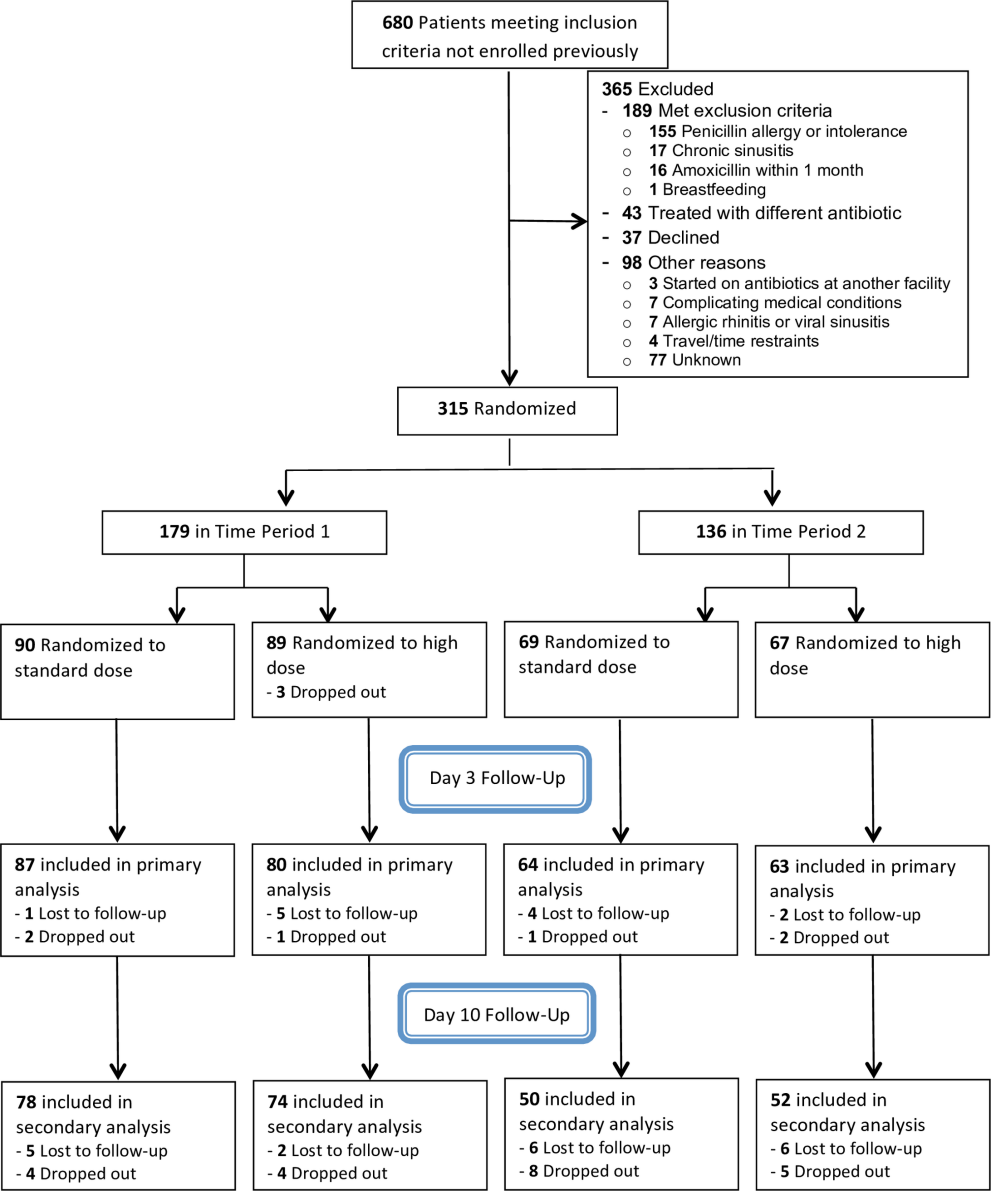
CONSORT flowchart of study participants. Reasons for exclusion before randomization were mostly inferred from chart review. We had 30 dropouts during the course of the study. Reason for dropping out included failure to improve or worsening illness (12), adverse reaction (10), allergic reaction (2), fear of side effects (2), misplaced medication bottle (1), switched to different antibiotic for another infection (1), tablet size too large (1), self-discontinuation because of marked symptom improvement (1), and unknown (1). Some participants had multiple reasons for exclusion or for dropping out.

Fig 1 shows the flow of potential and actual participants. Of 680 eligible patients during the study period, we enrolled 315, the first 179 in Time Period 1 (November 18, 2014, through February 5, 2016) and the next 136 in Time Period 2 (February 6, 2016, through February 27, 2017). The most common reason eligible patients were not enrolled was allergy or intolerance to penicillin (155). We had dropouts during the study because of patient choice or inability to contact patients for follow-up (as shown in Fig 1), but we were able to assess the primary efficacy endpoint in 93% of participants. We stopped the trial because we had met our recruitment goal and needed to analyze the data before the primary authors finished their residencies.

The baseline characteristics of patients randomized to SD or HD amoxicillin/clavulanate over the entire study were not statistically different (Table 2) and did not differ between SD and HD in each time period except for smoking in Time Period 2 (2.9% of SD participants vs 13.4% of HD, P = 0.02) (Table 3). No participant had been hospitalized in the preceding month.

**Table 2:** Baseline characteristics of participants as a whole by dose.

	Standard Dose	High Dose	P-value
Total (No. [%])	159 (50.5%)	156 (49.5%)	0.94
Age in years (mean ± SD)	48.0 ± 15.9	45.9 ± 14.8	0.22
Gender			0.55
*Male* (No. [%])	44 (27.7%)	48 (30.8%)	
*Female* (No. [%])	115 (70.8%)	108 (69.2%)	
Active tobacco use (No. [%])	19 (12.0%)	20 (12.85)	0.82
COPD/asthma (No. [%])	29 (18.2%)	30 (19.2%)	0.82
Allergic rhinitis (No. [%])	82 (51.6%)	80 (51.3%)	0.96
Nasal steroid use* (No. [%])	34 (21.4%)	31 (19.9%)	0.74
Antibiotic use in past month (No. [%])	2 (1.3%)	4 (2.6%)	0.45
Diabetes (No. [%])	16 (10.1%)	15 (9.6%)	0.89
Heart disease (No. [%])	11 (6.9%)	8 (5.1%)	0.51
History of sinusitis (No. [%])	63 (39.6%)	60 (38.5%)	0.83
Days of illness (mean ± SD)	14.8 ± 8.8	14.3 ± 8.1	0.55
Sinusitis category** (No. [%])			0.78
1) Duration ≥ 10 days	101 (63.5%)	103 (66.0%)	
2) Severe symptoms	18 (11.3%)	19 (12.2%)	
3) Double sickening	40 (25.2%)	34 (21.8%)	
Mean Day 0 SNOT-16 item score*** (mean ± SD)	1.89 ± 0.56	1.99 ± 0.55	0.11

*Ascertained by chart review.

**Categories of sinusitis according to Infectious Diseases Society of American guidelines [2].

***Mean rating for each group of all ratings, on a severity scale from 0 to 3, of the 16 items of the SNOT-16 questionnaire (shown in Table 1).

**Table 3:** Baseline characteristics by Time Period and Dose.

	Time Period 1			Time Period 2		
	Standard Dose	High Dose	P-value	Standard Dose	High Dose	P-value
Total (No. [%])	90 (50.3%)	89 (49.7%)		69 (50.7%)	67 (49.3%)	
Age (years ± SD)	47.4 ± 15.3	46.8 ± 14.5	0.80	48.8 ± 16.6	44.6 ± 15.3	0.13
Gender:			0.91			0.28
Male (No. [%])	29 (32.2%)	28 (31.5%)		15 (21.7%)	20 (29.9%)	
Female (No. [%])	61 (67.8%)	61 (68.5%)		54 (78.3%)	47 (70.2%)	
Active tobacco use (No. [%])	17 (18.9%)	11 (12.4%)	0.23	2 (2.9%)	9 (13.4%)	0.02
COPD/asthma (No. [%])	19 (21.1%)	18 (20.2%)	0.88	10 (14.5%)	12 (17.9%)	0.59
Allergic rhinitis (No. [%])	46 (51.1%)	45 (50.6%)	0.94	36 (52.2%)	35 (52.2%)	0.99
Nasal steroid use* (No. [%])	21 (23%)	16 (17.9%)	0.38	13 (18.8%)	15 (22.4%)	0.61
Antibiotic use in past month (No. [%])	2 (2.7%)	1 (1.1%)	1	0 (0%)	3 (4.5%)	0.12
Diabetes (No. [%])	10 (11.1%)	9 (10.1%)	0.83	6 (8.7%)	6 (9.0%)	0.96
Heart Disease (No. [%]d	9 (10.0%)	6 (6.7%)	0.43	2 (2.9%)	2 (3.0%)	0.98
History of sinusitis (No. [%])	39 (43.3%)	39 (43.8%)	0.95	24 (34.8%)	21 (31.3%)	0.67
Days of illness (No. ± SD)	15.2 ± 9.4	15.3 ± 9.3	0.94	14.3 ± 8.1	12.9 ± 6.1	0.23
Sinusitis category** (N [%])			0.60			0.59
1) Duration ≥ 10 days	61 (67.8%)	66 (74.2%)		40 (58.0%)	1. 37 (55.2%)	
2) Severe symptoms	9 (10.0%)	6 (6.7%)		9 (13.0%)	13 (19.4%)	
3) Double sickening	20 (22.2%)	17 (19.1%)		20 (29.0%)	17 (25.4%)	
Mean Day 0 SNOT-16 item score*** (mean ± SD)	1.89 ± 0.57	2.02 ± 0.54	0.12	1.89 ± .54	1.95 ± 0.55	0.521

*Ascertained by chart review.

**Categories of sinusitis according to Infectious Diseases Society of American guidelines [2].

***+ Mean rating for each group of all ratings, on a severity scale from 0 to 3, of the 16 items of the SNOT-16 questionnaire (shown in Table 1).

### Efficacy

As shown in Table 4, the primary outcome of a major improvement in symptoms—either “a lot better” or “no symptoms” at the end of Day 3—was reported overall by 36.4% of SD vs. 44.8% of HD participants (P = 0.15); during Time Period 1 by 37.9% of SD vs. 38.8% of ER HD participants (P = 0.91); and during Time Period 2 by 34.4% of SD vs. 52.4% of IR HD participants, an effect size of 18% (95% CI 0.75 to 35.26%, P = 0.04).

**Table 4:** Efficacy by Time Period and Dose.

			Standard Dose	High Dose	Effect size (95% CI)	P-Value
**Patients Reporting Major Improvement** * % (No.)	Day 3	Overall	36.42% (55/151)	44.76% (64/143)	8.33% (-2.94 to 19.60)	0.15
		Time Period 1	37.93% (33/87)	38.75% (31/80)	0.82% (-14.14 to 15.78)	0.91
		Time Period 2	34.38% (22/64)	52.38% (33/63)	18.01% (0.75 to 35.26)	0.04
	Day 10	Overall	76.56% (98/128)	78.57% (99/126)	2.01% (-8.79 to 12.74)	0.70
		Time Period 1	80.77% (63/78)	79.73% (59/74)	-1.04% (-12.44 to 14.63)	0.87
		Time Period 2	70.00% (35/50)	76.92% (40/52)	6.92% (-11.43 to 24.94)	0.43
Decrease in the Average SNOT 16 Item Score** Points on scale of 0–3	Day 3	Overall	0.81 pt.	0.91 pt.	0.10 pt. (-0.05 to 0.25)	0.19
		Time Period 1	0.75 pt.	0.87 pt.	0.12 pt. (-0.06 to 0.31)	0.19
		Time Period 2	0.91 pt.	0.97 pt.	0.07 pt. (-0.17 to 0.31)	0.58
	Day 10	Overall	1.34 pt.	1.45 pt.	0.11 pt. (-0.28 to 0.59)	0.20
		Time Period 1	1.32 pt.	1.48 pt.	0.16 pt. (-0.38 to 0.55)	0.14
		Time Period 2	1.37 pt.	1.40 pt.	0.03 pt. (-0.31 to 0.24)	0.806

*Percentage in each group reporting symptoms as “a lot better” or “no symptoms”

**The average decrease for each group in the mean item score on the SNOT-16 from baseline at Day 0 on a scale of 0–3. Minimally important difference = 0.5 [31].

The secondary efficacy outcomes did not differ significantly. Most patients in both arms reported major improvement at Day 10 regardless of time period. The mean SNOT-16 item scores from Day 0 did not improve significantly at either Day 3 or Day 10.

The only potential confounder that differed between groups, smoking status in Time Period 2, had no impact on Day 3 outcomes: among smokers, 3 of 9 taking HD in Time Period 2 had significant improvement vs. 1 of 2 taking SD (P = 0.66). Use of nasals steroids was associated with a trend toward a better outcome among those taking both SD and HD, but was not a confounder (see S4 Text: Impact of Nasal Steroid Use).

Since there was no difference between outcomes at Day 10, the day 30 data were of little value. We report them in S2 Table: Day 30 Results.

### Adverse effects

As shown in Table 5, the most frequent adverse effects were diarrhea (with or without abdominal pain) and, in women, vaginal discharge and itching. At Day 3, diarrhea was common, more so in HD than SD groups; diarrhea reported as severe (3 on the scale of 0–3) was uncommon except in IR HD patients (15.8% vs. 4.8%, an increase of 11.0%, 95% CI 0.1 to 21.8%, P = 0.05). Most diarrhea resolved by Day 10. Vaginal discharge and itching was also more common in HD than SD groups, especially in ER HD at Day 3 (19.6% vs. 4.6%, an increase of 15.0%, P = 0.01), but severe vaginal symptoms were uncommon. Abdominal pain without diarrhea was reported by only 23 participants (12 SD and 11 HD, P = 0.94) and rash by only 6.

**Table 5:** Adverse effects by Time Period and Dose.

			Standard Dose	High Dose	Effect Size (95% CI)	P-Value
Diarrhea, Any* % (No.)	Day 3	Time Period 1	34.48% (30/87)	46.25% (37/80)	11.77% (-3.92 to 26.93)	0.12
		Time Period 2	30.65% (19/62)	47.37% (27/57)	16.72% (-1.94 to 34.32)	0.06
	Day 10	Time Period 1	16.67% (13/78)	28.38% (21/74)	11.71% (-2.45 to 25.58)	0.08
		Time Period 2	12.00% (6/50)	17.31% (9/52)	5.31% (-9.98 to 20.32)	0.45
Diarrhea, Severe* % (No.)	Day 3	Time Period 1	5.75% (5/87)	7.50% (6/80)	1.75% (-5.87 to 9.38)	0.65
		Time Period 2	4.84% (3/62)	15.79% (9/57	10.95% (0.10 to 21.80)	0.05
	Day 10	Time Period 1	1.25% (1/80)	5.33% (4/75)	4.08% (-2.65 to 11.94)	0.15
		Time Period 2	0.00% (0/51)	3.85% (2/52)	3.85% (-3.90 to 13.22)	0.16
Vaginal Itching or Discharge, Any % (No.)	Day 3	Time Period 1	4.62% (3/65)	19.64% (11/56)	15.02% (2.48 to 28.32)	0.01
		Time Period 2	7.84% (4/51)	15.91% (7/44)	8.07% (-6.34 to 23.32)	0.22
	Day 10	Time Period 1	15.79% (9/57)	23.08% (12/52)	7.29% (-8.74 to 23.37)	0.34
		Time Period 2	17.50% (7/40)	23.68% (9/38)	6.18% (-13.40 to 25.61)	0.50
Vaginal Itching or Discharge. Severe % (No.)	Day 3	Time Period 1	0% (0/65)	0% (0/56)	0	1.00
		Time Period 2	1.96% (1/51)	2.27% (1/44)	0.31% (-8.46 to 10.25)	0.92
	Day 10	Time Period 1	5.26% (3/57)	3.85% (2/52)	1.41% (-8.84 to 11.36)	0.73
		Time Period 2	2.56% (1/39)	0.00% (0/37)	2.56% (-7.25 to 13.47)	0.33

*Diarrhea with or without abdominal pain.

### Additional analyses

At Day 10, 91.3% of SD and 90.5% of HD participants reported taking all 14 doses of antibiotics (P = 0.83). The percent unwilling to take the antibiotic again did not differ significantly after treatment with SD vs. HD (10.5% vs. 15.1%, P = 0.28) (Table 6).

**Table 6:** Reasons for not taking antibiotic again*.

	Time Period 1			Time Period 2		
	Standard dose N = 75	High dose N = 74	P-value	Standard dose N = 49	High dose N = 52	P-value
Number who would not take (% of total)	8 (10.7)	11 (14.9)	0.44	5 (10.2)	8 (15.4)	0.44
Severe diarrhea	1 (12.5)	2 (18.2)		0 (0)	4 (50.0)	
Any side effect	4 (50.0)	7 (63.6)		2 (40)	8 (100)	
Lack of improvement	3 (37.5)	3 (27.3)		1 (20)	1 (12.5)	
Unclear	1 (12.5)	1 (9.1)		2 (40)	0 (0)	

*Reasons inferred from chart review.

### Bacterial colonization

Among the 231 anterior nasal cultures (Table 7), only 11 grew *S*. *pneumoniae*, none resistant to penicillin (although resistance may not have been tested in 3). Only 4 of the 38 cultures growing *Staphylococcus aureus* were resistant to methicillin. Thus, at most 7 of the first 231 patients were colonized with pathogens resistant to amoxicillin/clavulanate.

**Table 7:** Bacterial colonization*.

Culture Report	Number (Percent)
No growth or normal flora	139 (60)
Methicillin-sensitive *Staphylococcus aureus*	32 (14)
*Haemophilus influenzae*	25 (11)
*Streptococcus pneumoniae*	9 (4)
*Moxarella catarrhalis*	9 (4)
*Staphylococcus epidermidis*	7 (3)
Methicillin-resistant *Staphylococcus aureus*	5 (2)
Other	5 (2)

*Cultures of the anterior nares of the first 231 patients

## Discussion

Primary care patients with clinically-diagnosed acute bacterial sinusitis had a markedly greater improvement in symptoms after 3 days of treatment with an IR formulation of HD amoxicillin/clavulanate than with SD—an increase from 34.4% to 52.4% in those reporting symptoms “a lot better” or “no symptoms” (a number-needed-to-treat of 6). Patients taking the ER formulation had an increase only from 37.9% to 38.8%.

This difference in efficacy was unexpected. One explanation might be a difference in patient populations. Patients taking SD vs. HD in the two time periods were very similar except for a larger percentage of smokers among those taking IR HD, but this had no impact on relative outcomes. It would seem hard for other, unmeasured differences to explain the large difference in efficacy. The more likely explanation is the pharmacokinetic and pharmacodynamic differences in the two formulations. Since the absorption of clavulanate in combined formulations is independent of the absorption of amoxicillin [34–35], the key is likely the difference between the HD amoxicillin formulations. Even though amoxicillin absorption is non-linear, ER provides elevated serum levels for longer periods of time, while IR reaches higher peak levels [36]. It is possible that, for acute sinusitis, high peak serum levels are needed to achieve adequate amoxicillin concentrations in the inflamed, fluid-filled sinus cavities. This would fit with the data on acute otitis media in children [6–8].

This implication about treatment should, however, be qualified. First, the improvements in the SNOT-16 scores were neither clinically nor statistically significant. In designing the study, we considered the overall judgment of improvement by the patient a clinically more important—and possibly more valid [25]—endpoint than the change in a composite score of multiple different symptoms. The data collectors found that participants gave improvement ratings quickly and confidently. In measuring health-related quality of life, however, researchers continue to debate the relative validities of an overall judgment vs. a sum of separate judgments and of assessing improvement directly vs. inferring it from changes in assessments of current states [25,37–43]. Second, the IR HD caused a considerable increase by Day 3 in diarrhea perceived as severe—15.8% vs. 4.8% (a number-needed-to-harm of 9). Studies of ER HD have, overall, found slightly higher rates of diarrhea with ER HD than with lower doses—11.5% vs. 11.9% [3], 12.4% vs. 8.6% [21], 16.7% vs. 14,4% [22], and 16.5% vs. 13% [23]—but with very few cases reported as severe. No data exists on IR HD, but the intestinal flora or other aspects of the gastrointestinal tract may be sensitive to the IR’s higher peaks. By Day 10, however, most severe diarrhea (and other adverse effects) had resolved. Third, as we expected, by Day 10, most patients felt very much improved or without symptoms, with no significant differences among patients treated with HD vs. SD in either time period. Fourth, the percent of patients at Day 10 who, after experiencing adverse effects, said they would not take the antibiotic again was higher for both HD formulations (although not significantly so) and even included a few patients who had major improvement at Day 3. Patients may place less weight on achieving the improvement they expect from any antibiotic than on suffering adverse effects they attribute to this particular antibiotic. Fifth, our findings may not be applicable to areas with a high prevalence of resistant bacteria since the prevalence in our patients was very low. The findings would, however, be applicable to most outpatient practices.

The study has multiple limitations. First, owing to the need to change the HD formulation midway through the trial when the study medication became unavailable, the power of each time period was diminished. The important impact of the IR formulation of HD should not be disregarded [44], but needs to be confirmed. Second, the original effort to collect information on patients treated for sinusitis outside of the study had to be abandoned as an unnecessary burden in a busy primary care practice. We had to perform a retrospective chart review of those diagnosed with acute sinusitis to ascertain the reasons for non-participation. Third, the clinicians no doubt enrolled some patients who did not have true bacterial sinusitis and, by decreasing the number who might respond quickly to antibiotics, reduced the differences between the SD and HD groups. Fourth, all follow-up calls were made by the 4 residents and 5 medical students who were busy with clinical rotations and occasionally had trouble contacting participants. Fifth, we did not advise participants to start taking nasal steroids although their use has been shown to improve symptoms [45] and is recommend by the IDSA [2]. We did not ask participants about current nasal steroid use at enrollment and, therefore, had to infer its use from a subsequent review of the electronic record of the enrollment office visit. We determined that use was not a confounder, but cannot be certain that all patients were actually taking the steroids listed in their health record. Sixth, we did not ask participants at days 3 and 10 if they had irrigated with nasal saline (as suggested in our handout to enrollees). Whether nasal saline irrigation actually improves symptoms significantly is, however, still uncertain [46].

Our findings need, of course, to be replicated. In particular, both the impact on patients of IR HD amoxicillin/clavulanate and the use of the global rating of sinus symptom improvement as a primary outcome need further investigation (as we are now doing). In the meantime, however, we make the following recommendations. First, clinicians should consider offering IR HD amoxicillin/clavulanate—amoxicillin/clavulanate 875/125 plus amoxicillin 875 mg twice a day for 7 days—to adults with sinusitis defined in accordance with IDSA criteria. They can consider this, contrary to IDSA guidelines [2], even if they know or suspect that the prevalence of penicillin-resistant pneumococci is low (as in our study population). Second, they should highlight the increased likelihoods of both more rapid improvement (and, therefore, the ability to resume normal activities) and severe diarrhea, so that patients can choose which is of greater importance. Patients with a pressing need to return to work or school might opt for IR HD; those who prefer a lower risk of diarrhea might choose either SD or another antibiotic. Third, clinicians should stress that benefits and detriments are largely short-term, with most patients getting better by Day 10 regardless of treatment.

## Supporting information

S1 Dataset Sinusitis study. (XLSX)

S1 Table Consort checklist for acute sinusitis study. (DOC)

S2 Table Day 30 results. (DOCX)

S1 Text Original protocol for acute sinusitis study. (DOC)

S2 Text Revised protocol for acute sinusitis study. (DOC)

S3 Text Suggestions for treating sinusitis symptoms. (DOCX)

S4 Text Effect of nasal steroid use. (DOCX)
