# Supplementary material for: High-dose versus standard-dose amoxicillin/clavulanate for clinically-diagnosed acute bacterial sinusitis: A randomized clinical trial
Source: PLoS One. 2018 May 8;13(5):e0196734. doi: 10.1371/journal.pone.0196734 (PMC5940197; doi:10.1371/journal.pone.0196734)
Supplement: S2 Table — (DOCX) [file pone.0196734.s003.docx]

**S2 Table: Day 30 Results**

A. Efficacy by Dose

1) Patients Reporting Major Improvement at Day 30 (% [No.])

|  | Standard Dose | High Dose | P-value |
| --- | --- | --- | --- |
| Overall | 87.3%  (103/118) | 89.4%  (104/119) | 0.98 |
| Time Period 1 | 90.0%  (63/70) | 84.9%  (62/73) | 0.36 |
| Time Period 2 | 83.3%  (40/48) | 91.3%  (42/46) | 0.25 |

2) Decrease in Average SNOT-16 Item Score at Day 30 (No.)*

|  | Standard Dose | High Dose | Effect Size (95% CI) | P-value |
| --- | --- | --- | --- | --- |
| Overall | 1.5  (116) | 1.7  (119) | 0.19  (0.02 to 0.37) | 0.03 |
| Time Period 1 | 1.42  (69) | 1.65  (73) | 0.22  (-0.003 to 0.45) | 0.053 |
| Time Period 2 | 1.52  (47) | 1.67  (46) | 0.16  (-0.13 to 0.45) | 0.28 |

* Compared to score at enrollment. Minimally important difference = 0.5 [31]

B. Adverse Effects by Dose and Severity

1) Diarrhea at Day 30 (% of total taking SD or HD [No.])

| Severity (0-3) | 0 (none) | 1 | 2 | 3 |
| --- | --- | --- | --- | --- |
| SD | 94.9% (112) | 2.5% (3) | 1.7% (2) | 0.9% (1) |
| HD | 96.6% (115) | 3.4% (4) | 0% (0) | 0% (0) |

Overall P-value = 0.48 (by Fisher’s Exact)

2) Vaginal itching and discharge at Day 30 (% of total taking SD or HD [No.])

| Severity (0-3) | 0 (none) | 1 | 2 | 3 |
| --- | --- | --- | --- | --- |
| SD | 94.5% (86) | 1.1% (1) | 1.1% (1) | 3.3% (3) |
| HD | 93.3% (84) | 1.1% (1) | 2.2% (2) | 3.3% (3) |

Overall P-value 0.94 (by Fisher’s Exact)
