# Supplementary material for: High-dose versus standard-dose amoxicillin/clavulanate for clinically-diagnosed acute bacterial sinusitis: A randomized clinical trial
Source: PLoS One. 2018 May 8;13(5):e0196734. doi: 10.1371/journal.pone.0196734 (PMC5940197; doi:10.1371/journal.pone.0196734)
Supplement: S2 Text — (DOC) [file pone.0196734.s005.doc]

**S2 Text: Revised Protocol for Acute Sinusitis Study**

**Clinical trial of the treatment of acute sinusitis with standard-dose vs. high-dose amoxicillin/clavulanate**

Principal Investigator:

Paul Sorum, MD, PhD, Professor of Internal Medicine and Pediatrics

Co-Investigators:

Andrea Matho, MD, 2^nd^ year Med-Ped resident (the four 2nd-year residents will be responsible for the timely follow-up of enrolled patients)

Mary Mulqueen, MD, 2^nd^ year Med-Ped resident

Aaron Quidort, MD, 2^nd^ year Med-Ped resident

Miyuki Tanino, MD, 2^nd^ year Med-Ped resident

Sujata Kane, PA-C (who will be enrolling the most patients)

Christine McGovern, MS, PA-C (who will work with the residents to assure the smooth enrollment and complete follow-up of patients)

Joseph Wayne, MD, MPH, Associate Professor of Internal Medicine and Pediatrics

Gina Garrison, Pharm D, Associate Professor of Pharmacy Practice, Albany College of Pharmacy and Health Sciences (who will prepare the medications)

Sub-Investigators

The other attending physicians at the AMC Internal Medicine and Pediatrics office (who will be enrolling patients):

Rahim Dhanani, MD

Elizabeth Higgins, MD

Hamish Kerr, MD

Deborah Light, MD

Jennifer Lindstrom, MD

Tricia Pelnik-Fecko, MD

Ivelisse Verrico, MD

Kathleen Zabinski-Kramer, MD

The other Medicine-Pediatrics residents (who will be enrolling patients and making follow-up telephone calls)

Brady Bowen, DO

Melanie Ecung Deyss, MD

Laura El-Hage, MD

Sara Khalil, MD

Preetha Kurian, MD

Daniel Lavelle, MD

Manpreet Mann, MD

Lyndsay Molinari, MD

Uwa Otabor, MD

Samuel Park, MD

Britta Sundquist, MD

Lisa Thaler, DO

Others

Nursing staff (who will provide the patients with documents relating to the study)

Medical students for research experience or work-study (who will help the residents to follow-up telephone calls and who will, if possible, enter data into the Excel data base) (to be set up)

Statistical consultant: Michael Mulvihill, DPH, Einstein Medical Center, NYC

**A. Study Background and Purpose**

*Rationale*

The Infection Disease Society of America recommends, in its 2012 guideline on rhinosinusitis (IDSA, 2012), using amoxicillin/clavulanate regular dose (875/125 bid) as first-line treatment for acute sinusitis in adults. It recommends using high dose (2000/125 bid) if the risk of penicillin-resistant S. pneumonia in the community is greater than 10%. But what should a clinician prescribe if, as in most communities, the clinician does not know the rate of penicillin resistance?

*Background*

Studies in adults have repeatedly demonstrated that treatment of clinically-defined acute sinusitis with conventional doses of amoxicillin (Williamson, et al, 2007; Garbutt et al., 2012,), and even of amoxicillin/clavulanate (Wald et al, 1986; Bucher et al., 2003) provide little or no clinically significant benefit. How to account for the negative results?

First, antibiotics may not be useful, i.e. the persistence of symptoms may be a part of the natural history of acute sinusitis, and the patients’ immune systems are adequate to eradicate the bacteria. This is the conclusion, for example, of Garbutt and colleagues (2012).

Second, the studies were small, and several other factors are likely to have reduced their statistical power to find a clinical benefit if there was one. 1) The placebo effect may be so strong that, especially in studies with largely subjective endpoints (the patients’ ratings of symptoms), it may obscure any true impact from killing bacteria with antibiotics (e.g., Wechsler et al, 2011). 2) The studies contained many people without true bacterial sinusitis (i.e., who had symptoms that qualified them but who did not actually have bacteria as the cause). In one study, patients with pathogens in their nasophargneal secretions improved faster if given antibiotics and those without pathogens did not (Kaiser, et al., 2001). But treating people without pathogens in the sinuses is unavoidable in clinical practice since clinicians must rely largely on non-specific symptoms and signs. 3) The sickest patients—those most likely, it would seem, to benefit from antibiotics--were excluded (and treated with antibiotics presumably), thereby limiting the study to those whose benefit would be both less likely and less dramatic (whose change in symptom scores could not be as great). Yet it is precisely this group that it is important, and ethically justifiable, to study. 4) The overall benefit was diminished by those infected with bacteria resistant to the antibiotic. In a meta-analysis of studies in 1990-2006 (Payne & Benninger, 2007), the culture rates were 32.7% for S. pneumoniae, 31.6% for H. influenzae, 10.1% for S. aureus, and 8.8% for M. catarrhalis. Thus a substantial proportion of patients had positive cultures for bacteria that would not be much affected by amoxicillin (see also Sokol, 2001; Ehrhardt & Russo, 2001 Jacobs et al., 2004). Accordingly, the Infectious Disease Society of American in its 2012 guidelines recommends regular-dose amoxicillin/clavulanate (rather than amoxicillin alone) as first-line treatment (IDSA, 2012), even though, as mentioned above, the clinical benefit of even amoxicillin/clavulanate is in question (Wald et al, 1986; Bucher et al, 2003). One reason for the questionable value of amoxicillin/clavulanate may be that clavulanate has been shown (Dinis et al, 2000) to have uneven penetration into sinus tissue (and would likely, therefore, to have at least as uneven penetration into sinus fluid). Moreover, the impact of adding clavulanate is diminished, as acknowledged by the IDSA, by the presence, in a variable proportion of patients, of penicillin-resistant S. pneumoniae (as well as methicillin-resistant S. aureus); it is for this reason that the IDSA recommends high-dose amoxicillin/clavulanate when the rate of penicillin-resistant S pneumoniae exceeds 10%. Indeed, the superior effectiveness of high dose was suggested by a small retrospective study in which the number of resistant S. pneumoniae in nasopharygeal swabs was reduced from 8 cases before treatment to 5 cases after treatment with 1.5 grams per day of amoxicillin with clavulanate but from 8 cases to only 1 case after treatment with 4.0 grams day of amoxicillin with clavulanate (Brook et al., 2005).

Third, studies could have been falsely negative if the methods used to measure the effect underestimated it. Garbutt et al (2012) used the mean response to the 16 questions of the SNOT-16 as the primary outcome. Is it possible that the responses to the truly meaningful questions (e.g. fatigue) were diluted by responses to questions that are less important to the patient or do not change very fast (e.g. runny nose) or are not much affected by antibiotics (e.g. sneezing)? In support of its validity as a measure of treatment effect, however, the SNOT-16 was validated by another group (Quadri et al, 2013), and the question on subjective improvement also showed no significant difference at day 3 (37% of amoxicillin group vs 34% of placebo group reported that symptoms were a lot better or absent), although at day 7 there was a significant increase in subjective improvement in the antibiotic group (74% vs 56%, p=.02).

Fourth, the conventional dose of amoxicillin—500 mg tid or 875 mg bid--may be inadequate, i.e. may not reach concentrations in the sinus cavities sufficient to kill the bacteria (Carenfeld et al., 1975). The issue in this fourth potential cause of previous negative results is three-fold. 1) Diffusion of antibiotic into a closed space is difficult, so that concentrations in the sinuses are likely--for some antibiotics, and in particular for amoxicillin--to be lower in the sinus cavities than in the blood (or in the sinus mucosa). In one study, penicillin was measurable in the secretions only if the serum concentration was high, but in contrast doxycline concentrations were measurable in both mucosa and secretions (Eneroth et al, 1975). This decrement may increase, at least for some antibiotics, as the membranes become thickened, i.e., as the infection becomes more subacute and chronic. For example, on day 10 of therapy in a study comparing amoxicillin and cyclacillin, mean concentrations of both drugs in serum were 2.5 to 2.7 micrograms/ml, but no antibiotic was detectable in 20 of 21 simultaneous sinus aspirates (Scheld et al, 1986), and azithromycin concentrations the day after the last dose were 2.33 micrograms per ml in acute sinusitis patients and 0.38 in chronic sinusitis patients (Karma et al). In contrast, in chronic sinusitis patients undergoing sinus surgery, concentrations of moxifloxacin were greater in sinus mucosa than in the blood (presumably because of inflammation), although sinus aspirate tests were not done (Gehanno et al, 2002). The antibiotic needs to get into the sinus cavities, not just to the sinus mucosa. 2) In purulent secretions, the pO2 is close to zero and the pCO2 is higher than in venous blood, which impedes the effect of antibiotics (Carenfelt & Lundberg, 1976). Higher concentrations might, therefore, be needed to get the same effect. 3) As mentioned earlier, strains of S. pneumoniae have become increasingly resistant to penicillin or amoxicillin because of a greater difficulty of its getting through the bacterial cell walls. The result is that higher concentrations of amoxicillin must be present in the sinus cavities to force enough amoxicillin through these cells walls to inhibit growth and cause cell death.

The study by Garbutt et al (2012) required symptoms for 7-28 days except in cases < 7 days with initial improvement and subsequent worsening. In fact, 143 of 166 (88%) were in the 7-28 day category; the mean duration of symptoms for the amoxicillin group was 11.2 days (SD 5.7) and median 10.0 (interquartile range 7.0-14.0) and was almost identical for the placebo group. Thus many of the participants had a long duration of symptoms and, therefore, a likely increased difficulty for antibiotics to diffuse through the thickened sinus membranes. The prevalence of resistant S. pneumoniae seems, however, to have been low. The S. pneumoniae in the children of the community had a prevalence of nonsusceptibility to amoxicillin of only 5% (1 out of 21 nasal isolates) in 2004 (Garbutt et al., 2006). Although this finding was among children recently vaccinated with up to 3 doses of pneumococcal conjugate vaccine, it seems likely that S. pneumoniae susceptibility in adults would parallel that in children and would be unlikely to have increased greatly over the 2 years before enrollment started in their sinusitis study (especially since the percentage of children with 3 doses of vaccine was increasing). It is not clear, therefore, whether the amoxicillin concentrations in the sinus cavities of a significant number of adults in the study by Garbutt et al would have been inadequate to treat S. pneumoniae.

Studies in children with acute otitis media and acute sinusitis provide support for the concern that conventional doses of amoxicillin (whether alone or with clavulunate) may not reach concentrations sufficient to treat many pneumococci as well as other pathogens. First, through tympanocentesis, researchers described the decrement in concentrations of antibiotics in the middle ears of children with acute otitis media (e.g. Nelson et al, 1994). These studies have not, to our knowledge, been done in sinuses (or in abscesses in other locations), but a similar decrement would be expected since the sinuses are also cavities and drainage is impeded (even if they are not usually fully closed off). This evidence was felt to be strong enough to prompt a revision of the treatment guidelines for acute otitis media in children even before there was any clinical demonstration of the superiority of higher doses of amoxicillin. The new guideline raised the daily dose of amoxicillin from 40 to 80-90 mg per kg and advised giving it in two rather than three divided doses. As a result, a child with acute otitis media weighing 40 pounds gets the same daily dose of amoxicillin as an adult with sinusitis several times her weight. Second, while the studies on acute otitis media have provided only limited confirmation of the new dosing of acute otitis media (Garrison et al, 2004), a recent study (Wald et al, 2009) on children age 1-10 years with clinical sinusitis showed a marked benefit of high-dose amoxicillin-clavulanate (with amoxicillin at 80-90 mg/kg) vs. placebo: 50% of those receiving antibiotic were cured and only 14% of those receiving placebo (and only 14% vs. 68% had treatment failure) There is no reason to believe that adults with acute sinusitis differ fundamentally from school-age children.

The IDSA recommends that high-dose amoxicillin-clavulanate be prescribed when the rate of penicillin-resistant S. pneumonia exceeds 10%. This recommendation is, however, difficult to follow in clinical practice. How are clinicians to know what the community rate of resistance is? In Albany, NY, cultures in 2013 (and earlier) in the ED, the adult and children’s wards, and the intensive care units each showed over 90% sensitivity to penicillin (Sarah Elmendorf, MD, personal communication, January 13, 2014) but it is uncertain to what extent these figures predict community resistance patterns. Even prior antibiotic use—known to select resistant bacteria (Sokol, 2001)—does not necessarily increase colonization with penicillin-resistant S. pneumoniae (Varon et al, 2000). In addition, how can clinicians, at least primary care clinicians, know what kind of bacteria (if any) is present in their patients’ sinuses? They cannot in their offices do sinus aspirations (the gold standard) or endoscopically-guided middle meatal cultures (Joniau et al., 2005), and they cannot rely on nasopharyneal cultures (unlike rapid tests and cultures for strep throat) because they are expensive; they give results only after, in many cases, a treatment decision must be made; and they do not reliably indicate what pathogen (if any) is causing the sinusitis (Benninger et al, 2002), even if it appears that colonization of the nasopharynx with a pathogen occurs prior to its infecting the sinuses (Han et al, 2011). Furthermore, it is possible that higher levels of amoxicillin in the sinuses will hasten clinical improvement even in patients without penicillin-resistant S. pneumonia. Thus the rate of colonization of the nasopharynx with penicillin-resistant S pneumonia may have little effect on the rate of symptom resolution with antibiotic treatment.

*Purpose of Current Study*

The purpose of the study is, using study procedures duplicating in large part those of Garbutt et al (2012), to compare the benefits of 2 treatment arms—high-dose amoxicillin/clavulanate (2000 mg amoxicillin/125 mg po bid) vs regular-dose amoxicillin/clavulanate (875 mg amoxicillin/125 mg po bid)--at day 3 (primary endpoint) and day 10 (secondary endpoint), by reported degree of improvement (primary endpoint, as in Wald et al., 2009) and by SNOT-16 (secondary endpoint, but which was the primary endpoint in Garbutt et al, 2012). We will use amoxicillin/clavulanate, rather than amoxicillin alone, to be in accord with the IDSA 2012 guideline (IDSA, 2012). The duration will be 7 days in line with the IDSA guidelines. We will not have a pure placebo arm since 1) the IDSA guidelines recommend treatment; 2) clinical studies (Wald et al, 1986; Bucher et al., 2003) suggest that treating with the IDSA-recommended regular-dose amoxicillin/clavulanate is equivalent, or almost equivalent, to giving a placebo; 3) we will not exclude patients who we would not want to risk giving a true placebo; and 4) we know that, because of patients’ continued belief in the efficacy of antibiotics, recruitment will be easily in a study with two treatment arms, rather than treatment and placebo arms. In addition, we will study whether the response to antibiotics can be predicted by the presence or not of penicillin-resistant S. pneumoniae in the nasopharynx.

Thus the study questions are: 1. Is high-dose amoxicillin/clavulanate superior to regular-dose in improving the clinical symptoms of presumed sinusitis? 2. Does the higher dose result in increased incidence and/or severity of adverse effects, such as diarrhea? 3. Does the resistance pattern of S. pneumonia in nasopharyngeal cultures predict response to antibiotic treatment? The hypotheses are yes for #1, no for #2, and no for #3 (but with the expectation of insufficient power to answer question #3).

**B. Study Design**

Study type: randomized, double-blind clinical study with two active treatment arms

Location: AMC Internal Medicine and Pediatrics office in Latham, NY

Duration: 2 years; each participant will be in the study for 30 days.

Subjects:

Inclusion criteria

1. Adults age 18 and over. The study on children already indicates a benefit of high-dose amoxicillin-clavulanate over placebo. The restriction to adults eliminates the need for parental consent.
2. Presenting with symptoms consistent with acute sinusitis according to standard guidelines. Garbutt et al, 2012, used: a) history of maxillary pain or tenderness in the face or teeth, purulent nasal secretions, and rhinosinusitis symptoms for 7-28 days that were not improving or were worsening, or b) rhinosinusitis symptoms for <7 days that had significantly worsened after initial improvement. The IDSA’s 2012 guidelines are slightly different and will be used here:
   1. Persistent symptoms (as above) and not improving (lasting for > 10 days); or
   2. Severe symptoms or signs of fever > 102 degrees F and nasal discharge or facial pain (lasting for > 3-4 days); or
   3. Worsening symptoms or signs characterized by a new onset of fever, headache, or increase in nasal discharge following a typical viral URI that lasted 5-6 days and was initially improving (“double-sickening”).
3. Both the treating physician and the patient must think that antibiotic treatment, if it helped, would be indicated.

Exclusion criteria

1. Peviously enrolled in the study
2. Allergic or intolerant to amoxicillin, amoxicillin/clavulanate, ampicillin, or penicillin, or severe reaction to any other beta lactam.
3. Lactation, since no data on clavunlanate. Note that pregnancy is not exclusion since amoxicillin/clavulanate is category B.
4. Taking allopurinol since it increases substantially the risk of rash.
5. Cognitively unable to give informed consent and/or to provide accurate assessment of own symptoms (as assessed by the person obtaining consent).
6. Chronic or recurrent “sinus” problems. Defined as persistent symptoms of “sinus” congestion, not attributed to nasal allergies, for 8 weeks or more (Mayo Cinic, 2014) or 2 or more episodes of antibiotic-treated “sinusitis” in past 3 months. These patients are at risk of anatomical or immunological abnormalities and of harboring antibiotic resistant organisms.
7. Need to start high-dose amoxicillin/clavulanate, doxycycline, or levofloxacin (i.e. the clinician’s judgment that regular-dose amoxicillin/clavulanate would be unwise).
   1. Signs of severe infection (e.g. evidence of systemic toxicity with fever > 102 F)
   2. Amoxicillin or penicillin within the past month (not, as in the IDSA guidelines [2012], any antibiotics)
   3. Immunocompromised

Note that, unlike the IDSA guidelines, (2012) we do not plan automatically to treat with high-dose amoxicillin/clavunate all patients >65 or those who were hospitalized recently but not treated with antibiotics.

1. Suspicion of MRSA.
2. Drug warnings
   1. Chronic kidney disease with estimated GFR < 30;
   2. Hepatic impairment (not including isolated transaminase elevation <2 times normal);
   3. Current mononucleosis (because of amoxicillin-induced rash);
   4. History of antibiotic-associated colitis.
3. Need to hospitalize because of severity of infection, respiratory distress, or vulnerability of patient.

Treatment arms

1. Amoxicillin/clavulanate 1000/62.5 ER 2 tabs po bid for 7 days. (total pill burden 4 per day) OR, if ER becomes unavailable, amoxicillin/clavulanate 875/125 + amoxicillin 875 po bid for 7 days.
2. Amoxicillin/clavulanate 875/125 tabs 1 tab + 1 placebo tab po bid for 7 days (total pill burden 4 per day).

The duration of 7 days is in accordance with IDSA guidelines.

Both groups would be advised: 1) to instill saline into each nostril at least 2 times a day (1/4 tsp salt dissolved in 4 oz of distilled or boiled water, 1 teaspoonful administered into each nostril by sniffing, by syringe, or by Neti-pot; 2) to take acetaminophen for discomfort (not to exceed a total daily dose of 3 grams, or 2 grams if the patient drinks ETOH).

The pharmacist would prepare the medication bottles and randomize them (i.e., number the two sets of medication bottles as determined by a random number generator and keep the list of correlations between bottle number and actual contents).

Measurements (clinical)

1. Diagnostic criteria for acute sinusitis (as listed above): clinician to record these (including duration of illness and the category of acute sinusitis)
2. Initial additional data collection by nurse or clinician: previous side effects with antibiotics, antibiotics within past month, hospitalization in past 5 days, comorbidities (e.g., diabetes, heart disease, and lung disease)
3. Sinonasal Outcome Test-16 (SNOT-16) at days 0, 3, 10, and 30 (for validation of the form, see Garbutt et al, 2011, and Quadri et al, 2013).
4. Patients’ summary assessment of their condition at days 3, 10, and 30: a lot worse, a little worse, the same, a little better, a lot better, no symptoms (as used by Garbutt et al, 2012).
5. Side effects at days 3 and 10
   1. Diarrhea: none, mild, moderate, severe
   2. Allergic reaction: rash without itching, rash with itching or hives
   3. Other: abdominal pain, vaginal discharge and itching, other
6. Drop outs:
   1. The participant can exit the study at any time, if much sicker, by going to the emergency department
   2. The participant can request, at any time, treatment with open-label antibiotic, e.g. doxycycline or levofloxacin (recommended by the IDSA), at the discretion of the clinician in the office or on call.
   3. The participant will be formally given this option at the day 3 telephone call.
7. Participants will be asked additionally at day 10
   1. To guess which treatment they took (to evaluate adequacy of blinding, although it is expected that patients who get better faster will assume they took the higher dose).
   2. To indicate how many doses they took. We will not ask for the bottles to be returned (which would require an otherwise unnecessary office visit for the participants).
   3. To indicate if they would take that particular medication again if prescribed.
8. Participants will be asked additionally at day 30 if, and to what extent, they had persistence, resolution, or recurrence of symptoms.

Measurements (bacteriological)

Nasal cultures (anterior) will be performed on every patient. The cultures will be processed at the Albany Medical Center microbiology laboratory to assess:

1. What pathogens are present?
2. If S. pneumonia, what is its level of penicillin resistance?
3. If S. aureus, is it methicillin resistant?

Endpoints

1. Primary endpoint: Major mprovement of subjective symptoms at day 3 (i.e., 48-72 hours after starting treatment), rated as “a lot better” or “no symptoms.”
2. Secondary clinical endpoints:
   1. At least mild improvement in subjective symptoms at day 3: percent in each group rating their condition as “a little better,” “a lot better,” or “no symptoms”
   2. Improvement in SNOT-16 score at day 3. Garbutt et al. (2012) indicate that a minimally important difference is 0.5 (on a scale of 0-3), although they used an effect size of 0.25 for their study size determination (requiring a bigger sample size). We will compare the percent in each group with average improvement > 0.5.
   3. The same comparisons at days 10 and 30 (one month).
   4. Comparisons of side effects—diarrhea and others mentioned by participants on scale of 0=none to 3=severe--at days 3 and 10.
   5. Percent of who dropped out because of side effects or inefficacy (switched to alternative antibiotic or went to the ED).
   6. The impact of other clinical characteristics on the outcomes: age, sex, co-morbidities, sinusitis category.
3. Secondary culture endpoints
   1. The proportion of participants with S. pneumoniae on NP culture and the proportion of these with moderate and high resistance to penicillin
   2. The proportion of participants with moderately or highly resistant S penumoniae versus the others who achieve the primary and secondary clinical end points
4. Other questions
   1. What percentages were treated without meeting IDSA criteria; were excluded; and refused to participate?
   2. How frequently did participants guess correctly which medication they were taking, and were their guesses associated with outcomes?
   3. How did the change in SNOT-16 score—and individual questions—correlate with holistic ratings of improvement?
   4. How many patients had MRSA, and did carriage of MRSA affect outcomes?

Sample size determination

1. For improvement in symptoms at day 3: Garbutt et al, 2012, has 34% symptom improvement in controls (placebo); if a clinically meaningful impact of high-dose amoxicillin-clavulanate is to increase the response rate to 50% (giving a NNT of 7), 146 patients in each group would be required to have an 80% power to detect this difference (if it exists), with alpha .05 (<http://www.sealedenvelope.com/power/binary-superiority/>) . The statistical consultant calculates, with alpha .05 only one-sided (not inappropriate since we are interested only in improvement), the needed sample would be 120 in each group.
2. For change in SNOT score: Garbutt et al, 2012 calculated that 100 patients in each group were needed to detect a true difference of .25. To detect the “clinically meaningful difference” of .50, fewer would be needed.

We propose, therefore, to aim for 150 patients in each group, i.e. a total of at least 300 participants in Med-Ped over a 2 year period.

In our patient population of over 10,000, we see about 10 patients a week with presumed acute sinusitis during the non-summer months—a total of over 600 last year with a diagnostic code of acute sinusitis--so we expect no problem in reaching our recruitment goal.

Procedure

Prior to enrollment

1. All clinicians achieve up-to-date certification in research ethics. Only those who are certified will enroll patients.
2. PI trains all the nurses and clinicians to be able to explain the study to patients, collect study data, and obtain informed consent.
3. Posters about the study are put in the waiting room and in the examining rooms.
4. The pharmacist prepares and numbers a set of medication bottles with either standard or high dose amoxicillin/clavulanate based on random number generation. Each bottle will include a label with dosing directions. A study allocation list will be kept by the pharmacist in a secure place in the office. The medication bottles and a log sheet to record medication #, patient name, and date will be kept in the locked medication room.

Enrollment

1. The nurse gives every patient with respiratory symptoms a SNOT-16 form to fill out and a handout about the sinusitis study (the same text that is posted in the waiting room and the examining rooms.) The nurse helps the patient to fill out the SNOT-16.
2. The clinician then assesses every patient with respiratory symptoms for suitability for the study, i.e., determines if the patient has acute sinusitis and is eligible for study, using the standard diagnostic criteria and considering the inclusion and exclusion criteria (marking them on the Data Collection Form).
3. The clinician indicates the disposition of each patient treated for acute sinusitis: meets criteria for inclusion, but not asked to participate (e.g., lack of time); treated even though not meeting IDSA criteria for acute sinusitis; presence of exclusion criterion; meeting all criteria but declined to participate; or entered into the study.
4. The clinician or nurse explains the study based on the Enrollment Script; the clinician obtains informed consent; and the clinician or nurse makes a copy of the consent form to give to the patient.
5. The clinician or the nurse obtains a nasal culture. If the patient prefers, he or she can insert the swab (under supervision to make sure it goes to mid nasal passage).
6. The clinician or nurse obtains the already-randomized medication bottle, writes the patient’s name and date on the log sheet or (preferred) attaches a patient sticker, and writes the patient’s number on the informed consent and on the culturette. The clinician gives the bottle of medication to the patient.
7. The patient is told that one tab has regular amoxicillin with clavulanate, the other tab has either a different formulation of amoxicillin with clavulanate or else an inactive ingredient (so that patients are not surprised if the pills look slightly different).
8. The clinician or the nurse collects the completed SNOT-16 and gives the patient a handout with instructions on additional measures, in particular on nasal saline preparation and use and on acetaminophen dosing.
9. The clinician or nurse also gives the patient a copy of the questions that will be asked at the day 3 telephone call and requests the patient to fill it out at the specified time (between 48 and 72 hours after starting treatment) if, for any reason, the patient cannot be reached by telephone. If needed, the resident co-investigators will then arrange with the patient how to obtain this form.
10. The clinician fills out the Data Collection Form for day O. This and the SNOT-16 will be scanned into the EHR (they will serve as documentation of at least part of the office visit) and put into the study binder and kept in a locked drawer in the PIs office (clinicians will have access to the key).
11. The clinician enters into the EHR a diagnosis of acute sinusitis, a treatment of amoxicillin/clavulanate (without specifying the dose), and an indication in the office visit note that the patient is a study participant.
12. The nurse prepares the culturette in accordance with the procedure agreed on with AMC clinical pharmacology lab.

Follow-up

1. The resident co-investigators prepare, in collaboration with the residency coordinator and the residency team’s PA, a list of residents (or medical students) responsible for telephone follow-up each day. They make sure—through word-of-mouth, telephone calls, secure texting (using Cureatr), or secure AMC email—that the resident or medical student calls each patient at 3 days, 10 days, and 30 days and fills out the appropriate Data Collection Form. They may look into setting up a secure Google Group site where the lists can be kept and accessed. The resident co-investigators make sure that the completed Data Collection Forms for each patient are inserted into the patient’s study binder.
2. If the patient reports clinical worsening or bad side effects, the resident or student notifies the clinician on call (or, if more appropriate, the clinician at the office the next day).
3. If the participant calls at any time to report a clinical worsening or bad side effects, or if the participant reports this at the 3 days telephone call, the clinician involved can decide, along with the participant:
   1. To have the patient remain in the study, after reassurance and/or advice on treating side effects;
   2. To switch the patient to a different antibiotic because of apparent inefficacy or bad side effects (and drop her out of active part of study):
   3. To bring the patient into the office to be seen (and decide then if the patient will stay in or drop out of the study); or
   4. To send the patient to the ED (if quite ill) (and drop her out of the active part of the study).
4. If the participant drops out, the clinician can ask the pharmacists to look up the results of the culture (if done) to help guide further management. In practice, this is highly unlikely since the patient who fails the study medication will be switched to another antibiotic (generally doxycycline or levofloxacin).
5. If a patient drops out, the clinician involved will fill out the Drop-Out form.
6. The participant who drops out of the active part of the study will still be called at 3, 10, and 30 days, if he or she agrees (to enable an intention to treat analysis with maximum data).
7. Culture results, identified only by the participant’s study number, will be collected by the pharmacist and kept separately. The results will be entered onto the Data Collection Form and the data base only at the end of the study, i.e. will not be known to the residents and students as they make phone calls. The pharmacist will tell a clinician the results for a particular patient only if the patient drops out and knowledge of the culture result might help in further management.
8. At the end of the study, the Data Collection Forms for each participant will be scanned into the EHR (so that the data will be clinically useful for the patient) and then destroyed (along with the Day 0 SNOT-16 form).

Costs

1. Medication
   1. Amox-clav 1000/62.5 two tabs bid x 7 days = $72.36 per patient. Total for 150 patents $10,854. We will try to get the antibiotic from 2 different generic manufacturers so that the 2 tabs look slightly different. Thus the total cost may be higher, but unlikely more than $12,000.
   2. Amox-clav 875/125 t tab bid x 7 days = $7.00 per patient. Total for 150 patients $1,050.
   3. Placebo. We are still trying to find information on costs, but doubt it will cost more than $5 per pill. Total for 150 patients $750 (or less).
   4. Packaging. Each patient will need 2 bottles and labels, likely $1 per patient. Total for 300 patients of $300.
2. Research assistant (coordinator if possible) (if the resident on medical home rotation or a work-study student is not a feasible option)
   1. Telephoning patients: 1 hour per patient x 300 patients @ $20 per hour = $6,000
   2. Entering data: 100 hours @ $20 per hour = $2,000.
3. Statistician: 20 hours @ $100 per hour = $2,000.
4. Cultures: estimate of $100 per culture= $30,000.
5. Duplication of forms: 600 @ 10 cents = $60.
6. Study binders: 300 @ 50 cents = $150.
7. Overall cost: $44,550.
8. Funds will come from the AMC Med-Ped Research Fund, derived from private contributions and held by the Albany Medical Center Foundation, at the discretion of the Medicine-Pediatrics Division Head (currently Dr. Jennifer Lindstrom).

**C. Subject Population**

Source of participants: the patients of AMC Internal Medicine and Pediatrics, 724 Watervliet-Shaker Drive, Latham, NY, who present with respiratory symptoms.

Total number of participants to be enrolled: 300.

Characteristics of population: adults, with no special populations (see Inclusion and Exclusion criteria above).

**D. Data Analysis**

See the Data Collection Sheet for a listing of the data that will be collected.

The Data Collection Sheets will be stored in a locked drawer in the PI’s office.

The data will be entered by the research assistant (or Med-Ped residents or medical students doing work-study) into an Excel data base stored on a password-protected computer in the PI’s office.

The Excel data base will be stripped of patient identifiers and transferred to SPSS by the statistical consultant, Dr. Mulvihill, who will, in turn, perform the analyses.

The primary analysis will be a simple chi-square analysis, with an alpha of .05%, of the difference in the primary endpoint (the proportion of participants who are “a lot better” or “no symptoms” at 3 days).

The analyses of the secondary outcomes (listed above) will depend on the nature of the data and be determined by the statistician.

If we must, because of medication shortages, switch the high dose arm from 1000/62.5 ER 2 bid to 875/125 + 875 bid, we will perform post-hoc tests of the impact of the switch on efficacy and side effects.

**E Risks**

Risks of treatment

The side effects of treatment with amoxicillin/clavunate include:

1. Allergic reaction, usually minor, sometimes severe enough to send to the ED, very rarely resulting in death. Wikipedia’s entry on anaphylaxis suggests death in about 1 out of 50,000 courses of penicillin.
2. Gastrointestinal troubles, primarily diarrhea, uncommonly C. difficile infection requiring hospitalization. The major culprit is clavunate, which is at the same dose in both treatment arms. Bucher et al (2003) found that odds ratio on day 7 of diarrhea, in those receiving standard dose amoxicillin vs. placebo was 3.89 abdominal pain, but did not give actual frequencies. Sethi et al. (2005) found a rate of diarrhea of 14.4% in those receiving standard dose amoxicillin/clavulanate.
3. In women, vulvo-vaginal Candidiasis, at a rate lower than diarrhea (Sethi et al. [2005]).
4. The recommended treatment is with standard-dose. The question is not whether high-dose amoxicillin, combined with clavunate, would cause any symptoms, but whether it would cause more symptoms than standard-dose. No difference was seen in a study of treatment of acute otitis media in children (Garrison, et al., 2004) and in the study of bronchitis in adults (Sethi et al, 2005). In the latter, the rates of diarrhea (the most common side effect) in high vs standard dose were 16.7% vs 14.4% (p = 0.36), and the rates of any adverse effect were 36.6% vs. 42.4%.
5. The percent of participants withdrawn because of adverse effects in Sethi et al. were 2.3% in the high dose group and 4.0% in the standard dose group.

Two other types of treatment risks are

1. Under-treatment, i.e., enrolling patients into the study who should have been sent to the ED or treated with high-dose amoxicillin/clavulanate or with levofloxacin. The clinician is required to make and document the judgment that the patient is not too sick. In addition, it will be far easier, in terms of clinician time and effort, to disqualify a patient (and to send him or her to the ED)
2. Over-treatment, i.e., enrolling patients who ought not to be treated. The clinician is required to document the findings justifying a diagnosis of acute sinusitis and to pick a diagnostic category. Again, the decision to enroll the patient will require much more time and effort than not enrolling the patient.

Risk of coercion

The patient may feel pressure from the clinician and nurse to enroll. The pressure is less likely because of the burden to the clinician if the patient is enrolled, but more likely if enrollment is proceeding too slowly.

Risk of loss of confidentiality.

The study will not be dealing with sensitive information. The efforts to protect patients’ identities and information are discussed below.

**F. Benefits**

The patients who are randomly assigned to high-dose amoxicillin/clavulanate may benefit from a faster rate of improvement and a higher cure rate.

Other patients will benefit from knowing if acute sinusitis should be routinely treated with high-dose rather than standard doses amoxicillin/clavulanate.

**G. Confidentiality**

Patient identifiers will be collected and stored. But we will make all efforts to prevent loss of confidentiality.

1. List of patient labels (or written names and dates of service) and study numbers will be kept in a secure location in the office; the Pharmacist will have access to it. This list will be filled out as a patient enters the study and receives a medication. This list can be used, if needed, at the end of the study (or during it if needed for a patient who drops out), to associate name/participant number with the treatment the participant received. It will be kept as a backup and check since this information should also be entered on the Data Collection Form and subsequently in the electronic data base. The list will be used at the end of the study to verify the accuracy of the data base (in terms of assignment to treatment arm) and then destroyed.
2. Data Collection Form for each participant will be kept in a locked drawer in the PI’s office; the clinicians (attendings, PAs, and residents) and the medical student research assistants will have access to it This form (shown in the Appendix) will have the patient’s name, age, MR #, and other clinical information. It will be used to enter these data into the electronic Excel data base. At the end of the study, the forms will be scanned into the patient charts (since the data may be useful clinically for the patients), the data in the Forms and in the data base will be cross-checked for accuracy, and the forms will be destroyed, i.e. about 2 years after the start of the study.
3. Culture reports. Cultures will be submitted to the laboratory with only the study number as an identifier; thus the results will come back with the study number as the only identifier. The reports will be kept in a separate folder in the locked drawer in the Pharmacy area along with the list of patents and study numbers. The pharmacist will be instructed to file the reports without looking at them. The reports will be examined only at the end of the study, unless (as mentioned above) there is an unforeseen need, because of patient deterioration, to know what grew in the culture.
4. Excel data base. The Excel data base will contain all the information listed on the Data Collection Forms. It will be kept in a password-protected computer in the PI’s office. The computer will not be connected to the Internet. The PI and the research assistant will have access to it. The data base will be preserved until the study is completed, the data have been analyzed, and planned publications have been submitted and accepted for publication, i.e. for up to 5 years after the start of the study (in case there are questions about the data or requests for further analyses). Then the data base will be destroyed.
5. SPSS data base. The Excel data base will be stripped of patient identifies (with the patient identified by study number) and transferred by the statistical consultant, Dr. Mulvihill, into SPSS for the statistical analyses. It will also be destroyed after 5 years.

**References**

Benninger MS, Appelbaum PC, Denneny JC, Osguthorpe DJ, Stankiewicz JA. Maxillary sinus puncture and culture in the diagnosis of acute rhinosinusitis: the case for pursuing alternative culture methods. Otolaryngol Head Neck Surg 2002;127:7-12.

Bucher HC, Tschudt P, Young J, Périat P, Welge-Lüssen, Hansjõrd Zũśt, Schindler C. Effect of amoxicillin-clavulanate in clinically diagnosed acute rhinosinusitis. A placebo-controlled, double-blind, randomized trial in general practice. Arch Intern Med 2003;163:1793-1798.

Carenfelt C, Eneroth CM, Lundberg C, Wretlind B. Evaluation of the antibiotic effect of treatment of maxillary sinusitis. Scand J Infect Dis 1975;7:259-264.

Carenfelt C, Lundberg C. Aspects of the treatment of maxillary sinusitis. Scand J Infect Dis Suppl 1976;82:78-81.

Chow AW, Benninger MS, Brook I, Brozek JL, Goldsteinr EJC, Hicks LA, Pankey GA, Seleznick M, Volturo G, Wald ER, File TM Jr. IDSA clinical practice guideline for acute bacterial rhinosinusitis in children and adults. Clin Inf Dis 2012;54 (15 April):e72-e112.

Critchley IA, Brown SD, Traczewski MM, Tilotson GS, Janjic N. National and regional assessment of antimicrobial resistance among community-acquired respiratory tract pathogens identified in a 2005-2006 U. S. faropenem surveillance study. Antimicrob Agents Chemother 2007;51:4382-4389.

Dinis PB, Monteiro MC, Martins ML, Silva N, Gomes A. Sinus tissue pharmcokinetics after oral administration of amoxicillin/clavulanic acid. Laryngoscope 2000;110:10-50-1055.

Ehrhardt AF, Russo R. Clinical resistance encountered in the respiratory surveillance program (RESP) study: a review of the implications for the treatment of community-acquired respiratory tract infections. Am J Med 2001;111 Suppl 9A:30S-35S.

Eneroth CM, Lundberg C, Wretlind B. Antibiotic concentrations in maxillary sinus secretions and in the sinus mucosa. Chemother 1975;21 Suppl 1:1-7.

Garbutt J, Snitznagel E, Piccirillo J. Use of the modified SNOT-16 in primary care patients with clinically diagnosed acute rhinosinusitis. Arch Otolarnygol Head Neck Surg 2011;137:792-797.

Garbutt JM, Banister C, Spitznagel E, Piccirillo JF. Amoxicillin for acute rhinosinusitis. A randomized controlled trial. JAMA 2012;307:685-692.

Garrison G, Sorum P, Hioe W, Miller M. High dose vs. standard dose amoxicillin for acute otitis media. Ann Pharmacopther. 2004;38:15-9.

Gehanno P, Darantière S, Dubreuil C, Chobaut JC, Bobin S, Pages JC, Renou G, Bobin F, Arvis P, Stass H. A prospective, multicentre study of moxifloxacin concentrations in the sinus mucosa tissue of patients undergoing elective surgery of the sinus. J Antimicrob Chemother 2002;49:821-826.

Han JK, Hendley JO, Winther B. Bacterial pathogens of acute sinusitis in the osteomeatal complex during common colds and wellness. Int Forum Allergy Rhinol 2011:1;356-360.

Jacobs MR, Bajaksouzian S, Windau A, Good CE, Lin G, Pankuch GA, Appelbaum PC. Susceptability of Streptoccous pneumonia, Haemophilus influenza, and Moraxella catarrhalis to 17 oral antimicrobial agents based on pharmacodynamic parameters: 1998-2001 U S Surveillance Study. Clin Lab Med 2004;24:503-530.

Joniau S, Vlaminck S, Van Landuyt H, Kuhweide R, Dick C. Microbiology of sinus puncture versus middle meatal aspiration in acute bacterial maxillary sinusitis. Am J Rhinol 2005;19:135-140.

Kaiser L, Morabia A, Stadler H, Ricchetti A, Auckenthaler R, Terrier F, Hirschel B, Khaw N, Lacroix JS, Lew D. Role of nasopharyngeal culture in antibiotic prescription for patients with common cold or acute sinusitis. Eur J Clin Microbiol Infect Dis 2001;20:445-451.

Karma P, Pukander J, Penttila M. Azithromycin concentrations in sinus fluid and mucosa after oral administration. Eur J Clin Microbiol Infect Dis 10:856-859.

Mayo Clinic. Available at <http://www.mayoclinic.org/diseases-conditions/chronic-sinusitis/basics/definition/con-20022039>. Accessed on May 25, 2014.

Nelson CT, Mason ED, Kaplan SL. Activity of oral antibiotics in middle ear and sinus infections caused by penicillin-resistant *Streptococcus pneumoniae*; implications for treatment. Pediatr Infect Dis J 1994;13:585-589.

Payne SC, Benninger MS. *Staphylococcus aureus* is a major pathogen in acute bacterial rhinosinusitis: a meta-analysis. Clin Infect Dis 2007;45:e121-127.

Quadri N, Lloyd A, Keating KN, Nafees B, Piccirillo J, Wild D. Psychometric evaluation of the Sinonasal Outcome Test-16 and activity impairment assessment in acute bacterial sinusitis. Otolaryngol Head Neck Surg 2013;149:161-167.

Scheld WM, Sydnor A Jr, Fatr B, Gratz JC, Gwaltney JM. Comparison of cyclacillin and amoxicillin for therapy for acute maxillary sinusitis. Antimicrob Agents Chemother 1986;30:350-353.

Sethi S, Breton J, Wynne B. Antimcrob Agents Chemother 2005;49:153-160.

Sokol W. Epidemiologiy of sinusitis in the primary care setting: results from the 1999-2000 respiratory surveillance program. Am J Med 2001;111 Suppl 9A:19S-24S.

Varon E, Levy C, De La Rocque F, Boucherat M, Deforche D, Podglajen I, Navel M, Cohen R. Impact of antimicrobial therapy on nasopharyngeal carriage of *Streptococcus pneumonia, Haemophilus influenza*, and *Brahhamella catarrhalis* in children with respiratory tract infections. Clin Infect Dis 2000;41:477-481.

Wald ER, Chiponis D, Ledsma-Medina J. Comparative effectiveness of amoxicillin and amoxicllin-clavunlate potassium in acute paranasal sinus infections in children: a double-blind, placebo-controlled. Pediatrics 1986;77:795-800.

Wald ER, Nash D, Eickhoff J. Effectiveness of amoxicillin/clavulanate potassium in the treatment of bacterial sinusitis in children. Pediatrics 2009;124:9-15.

Williamson IG, Rumsby K, Benge S, Moore M, Smith PW, Cross M, Little P. Antibiotics and topical nasal steroid for treatment of acute maxillary sinusitis. JAMA 2007;298:2487-2496.

**Appendix**

A. POSTER: In waiting room and in each examining room (and given to each patient with respiratory symptoms)

PLEASE SIGN UP FOR OUR ACUTE SINUSITIS STUDY

Our office is conducting a study to determine how to treat acute sinusitis most effectively.

Most respiratory symptoms are caused by viruses or allergies. Occasionally, however, people can develop bacterial infections in their clogged sinuses. Then it may be beneficial to take an antibiotic.

The Infectious Disease Society of America recommends treating acute bacterial sinusitis with amoxicillin (combined with clavulanate to make the amoxicillin more effective), except when patients are allergic to amoxicillin or penicillin.

The recommended dose of amoxicillin may, however, be too low to kill effectively the bacteria in your sinuses. This has been demonstrated for children, but not yet for adults. The purpose of our study is to see if a higher dose would benefit adults too.

We will know the answer only after—with your help—we complete the study!

B. SNOT-16 Questionnaire (to be given out by nurse when patient with respiratory symptoms is put in the room)

Rate how much you have been bothered, in terms of severity and frequency, by the following symptoms on a scale of 0=no problem, 1=mild or slight problem, 2=moderate problem, or 3=severe problem

1. Need to blow nose
2. Sneezing
3. Runny nose
4. Cough
5. Postnasal discharge
6. Thick nasal discharge
7. Ear fullness
8. Headache
9. Facial pain or pressure
10. Wake up at night
11. Lack of a good night’s sleep
12. Wake up tired
13. Fatigue
14. Reduced productivity
15. Reduced concentration
16. Frustrated, restless, or irritable

C. ENROLLMENT SCRIPT

I think (or, if the enrolled by the nurse, Sujata/Christine/Dr. X thinks) that you have a bacterial sinus infection and will benefit from treatment with amoxicillin, combined with clavulanate to make the amoxicillin more effective.

As you know, our office is conducting a study to determine if a higher dose of amoxicillin than that currently recommended by the Infectious Disease Society of America will be more effective without causing more side effects. We hope you will take part in the study.

If you agree to take part, we will call you in 3, 10, and 30 days to see how you are doing. If you have troubles with your infection or with the antibiotics, you should call us at any time. If you want to drop out of the study, you can do this at any time.

If you agree to take part, we will do a simple nasal swab to see what bacteria might be growing in your nose; we will not, however, know the results of the swab until after the study is done.

If you are interested in taking part, please read this Consent Form. I (or Sujata/Christine/Dr. X) will be back to see if you have any questions about it.

If you prefer not to participate, that is perfectly OK. I (or Sujata, Christine, or Dr. X) will treat you with the antibiotics recommended by the Infection Disease Society and acceptable to you.

D. SUGGESTIONS FOR TREATENT OF SYMPTOMS

1. For muscle or head aches, you can take acetaminophen (Tylenol). For safety, do not use more than 3,000 milligrams total of acetaminophen in 24 hours (including acetaminophen in combination with other prescribed or over-the-counter medications).
2. For thick nasal secretions, you should irrigate your nostrils with saline solution several times a day (as often as you need to keep your nose clear). This will provide relief and may also improve recovery by promoting drainage of your sinuses.
   1. Prepare saline by dissolving 1 teaspoon of kosher salt in 1 quart of boiled or distilled water (cooled to room temperature). You may use up to 2 teaspoons of salt if you find a stronger solution is more effective.
   2. Irrigate your nose with the saline solution by one of the following methods:
      1. Utilize a Neti-Pot or similar nasal irrigator. Follow its directions.
      2. Use a bulb or ear syringe. Fill it with saline, lean over the sink keeping your nose upright, place the tip of the syringe just inside one nostril pointing toward the middle (the nasal septum), gently but quickly squeeze the bulb until empty, allow to drain into the sink. Repeat 2-3 times in each nostril Gently blow your nose to clear it of remaining solution and mucous.
      3. If you cannot do these, fill a teaspoon with saline, occlude one nostril, sniff the saline up the other nostril, put your head back to allow it to seep to the back of your nose, do the same with your other nostril, and then blow your nose.

E. HANDOUT FOR DAY 3 TELEPHONE CALL

A member of our research team should contact you 48-72 hours after you start treatment in order to ask you the following questions. If, for any reason, our researcher does not get ahold of you before 72 hours, please write down your answers on this form We will then arrange with you how to obtain the form.

How are your sinus symptoms (scale of 1 to 6)? (circle one)

1 a lot worse, 2 a little worse, 3 the same, 4 a little better, 5 a lot better, or 6 no symptoms

SNOT-16

Rate how much you have been bothered, in terms of severity and frequency, by the following symptoms on a scale of 0=no problem, 1=mild or slight problem, 2=moderate problem, or 3=severe problem

1. Need to blow nose:
2. Sneezing:
3. Runny nose:
4. Cough:
5. Postnasal discharge:
6. Thick nasal discharge:
7. Ear fullness:
8. Headache:
9. Facial pain or pressure:
10. Wake up at night:
11. Lack of a good night’s sleep:
12. Wake up tired:
13. Fatigue:
14. Reduced productivity:
15. Reduced concentration:
16. Frustrated, restless, or irritable:

Do you have any side effects? (circle or check)

1. Diarrhea: 0 none, 1 mild, 2 moderate, 3 severe
2. Abdominal pain: 0 none, 1 mild, 2 moderate, 3 severe
3. (For women) vaginal discharge and itching: 0 none, 1 mild, 2 moderate, 3 severe
4. Rash: [ ] rash without itching [ ] rash with itching (or hives)
5. Other:

E. DATA COLLECTION FORM

See attached. (Each day—enrollment, day 3, day 10, and day 30--will be on a separate sheet of paper.)
