# Supplementary material for: High-dose versus standard-dose amoxicillin/clavulanate for clinically-diagnosed acute bacterial sinusitis: A randomized clinical trial
Source: PLoS One. 2018 May 8;13(5):e0196734. doi: 10.1371/journal.pone.0196734 (PMC5940197; doi:10.1371/journal.pone.0196734)
Supplement: S3 Text — (DOCX) [file pone.0196734.s006.docx]

**S3 Text: Suggestions for treating sinusitis symptoms**

Handout given to participants at enrollment.

SUGGESTIONS FOR TREATING SYMPTOMS

* For muscle or head aches, you can take acetaminophen (Tylenol). For safety, do not use more than 3,000 milligrams total of acetaminophen in 24 hours (including acetaminophen in combination with other prescribed or over-the-counter medications).

* For thick nasal secretions, you should irrigate your nostrils with saline solution several times a day (as often as you need to keep your nose clear). This will provide relief and may also improve recovery by promoting drainage of your sinuses.

1. Prepare saline by dissolving 1 teaspoon of kosher salt in 1 quart of boiled or distilled water (cooled to room temperature). You may use up to 2 teaspoons of salt if you find a stronger solution is more effective.
2. Irrigate your nose with the saline solution by one of the following methods:
   1. Utilize a Neti-Pot or similar nasal irrigator. Follow its directions.
   2. Use a bulb or ear syringe. Fill it with saline, lean over the sink keeping your nose upright, place the tip of the syringe just inside one nostril pointing toward the middle (the nasal septum), gently but quickly squeeze the bulb until empty, allow to drain into the sink. Repeat 2-3 times in each nostril. Gently blow your nose to clear it of remaining solution and mucous.
   3. If you cannot do these, fill a teaspoon with saline, occlude one nostril, sniff the saline up the other nostril, put your head back to allow it to seep to the back of your nose, do the same with your other nostril, and then blow your nose.
