# Supplementary material for: High-dose versus standard-dose amoxicillin/clavulanate for clinically-diagnosed acute bacterial sinusitis: A randomized clinical trial
Source: PLoS One. 2018 May 8;13(5):e0196734. doi: 10.1371/journal.pone.0196734 (PMC5940197; doi:10.1371/journal.pone.0196734)
Supplement: S4 Text — (DOCX) [file pone.0196734.s007.docx]

**S4 Text: Effect of Nasal Steroid Use**

Percent achieving the primary outcome (a global rating of “a lot better” or “cured” at the end of 3 days of treatment):

Overall: using nasal steroids 50.8% vs. not using 37.8%, p=0.06.

Among those using nasal steroids: SD 42.4% vs. HD 60.7%, p=0.15.

Among those not using nasal steroids: SD 34.8% vs. HD 40.9%, p=0.34.

Conclusion: nasal steroid use had an additive impact on the primary outcome, but was not a confounder.

Limitation: data on the use of nasal steroids was obtained not by asking the participant but from subsequent review of the electronic record of each enrollment visit.
